# Supplementary material for: Multidimensional Frailty and Vaccinations in Older People: A Cross-Sectional Study
Source: Vaccines (Basel). 2022 Apr 3;10(4):555. doi: 10.3390/vaccines10040555 (PMC9028390; doi:10.3390/vaccines10040555)
Supplement: Supplementary file 1 [file vaccines-10-00555-s001.zip › vaccines-1625606-supplementary.pdf]

Table S1. Prevalence, as percentages, of single domains values in the sample

Domain	Low risk=0	Medium risk=0.5	High risk=1	Missing
**ADL**	92.2	7.5	0.3	-
**IADL**	54.5	21.3	24.1	-
**Mobility**	84.0	13.5	2.5	-
**TYM score**	14.4	60.2	25.4	
**MNA**	55.5	27.6	7.8	9.1
**CIRS-SI**	5.6	31.3	63.0	-
**Number of medications**	33.2	36.7	21.3	8.8
**Living**	77.1	0.0	20.7	2.2

Abbreviations: ADL: activities of daily living; IADL: instrumental activities of daily living; TYM: test your memory; MNA: mini nutritional assessment; CIRS: cumulative illness rating scale, severity index; MPI: multidimensional prognostic index.
